# Patterns of interparental conflict and psychological distress among Australian mothers of autistic children

**DOI:** 10.1177/13623613251412202

**Published:** 2026-01-31

**Authors:** Alexis Kanat, Grace McMahon, Alison Fogarty, Rebecca Giallo, Monique Seymour

**Affiliations:** 1 Deakin University, Australia; 2 The Royal Children’s Hospital, Australia; 3 The University of Melbourne, Australia

**Keywords:** autism spectrum, interparental conflict, mental health, mothers

## Abstract

Mothers of autistic children often face unique emotional and financial challenges, contributing to heightened stress, poorer mental health and increased interparental conflict. This study examined interparental conflict over a 10-year period (child ages 4–14 years) among mothers of autistic children. The objectives were to: (1) compare interparental conflict levels between mothers of autistic and non-autistic children; (2) identify distinct interparental conflict trajectories among mothers of autistic children; and (3) examine the relationship between interparental conflict trajectories and mothers’ psychological distress when their children were age 14 years. Data were drawn from six waves of the Longitudinal Study of Australian Children. There were 333 mothers of autistic children and 8145 mothers of non-autistic children. Mothers of autistic children reported significantly greater interparental conflict than mothers of non-autistic children, peaking at child ages 4–5 years. Two interparental conflict trajectories were identified for mothers of autistic children: *consistently low* and *persistently elevated*, with the latter associated with greater psychological distress. These results highlight the importance of tailored support for mothers of autistic children to reduce interparental conflict and improve maternal well-being, ultimately strengthening overall family functioning.

**Lay Abstract:**

Mothers raising autistic children often deal with more emotional and financial stress than mothers of non-autistic children, which can lead to more frequent interparental conflict. This study looked at how interparental conflict changed over 10 years, from when children were 4 to 14 years old. Researchers analysed data from a large, longitudinal Australian study of children. They compared two groups: 333 mothers of autistic children and 8145 mothers of non-autistic children. The study aimed to: (1) examine differences in interparental conflict between the two groups, (2) identify distinct patterns of interparental conflict among mothers of autistic children and (3) explore how these patterns related to mothers’ psychological distress when their children were 14 years old. Mothers of autistic children experienced more interparental conflict than mothers of non-autistic children, particularly when their children were 4 to 5 years old. Two distinct interparental conflict patterns emerged among mothers of autistic children: one group experienced *consistently low* interparental conflict, while the other reported *persistently elevated* interparental conflict. Mothers in the *persistently elevated* interparental conflict group also reported greater psychological distress compared to those in the *consistently low* interparental conflict group. These results highlight the need for more personalised support for mothers of autistic children to help reduce interparental conflict and improve maternal well-being.

Many mothers view motherhood as the most rewarding experience of their lives (Apostolou & Kagialis, 2025; Nelson et al., 2014). However, its very significance can make it emotionally demanding and challenging (Chen, 2025; Kerr et al., 2020). These challenges are often pronounced when raising autistic children (Iao, 2024). For example, mothers of autistic children learn to navigate the core traits of autism, such as communication and social interaction difficulties (APA, 2022), while also contending with financial strain from costs related to specialised therapies, increased child care needs, advocacy and lifestyle changes (Anderson et al., 2020; Brewer, 2018; Dharanidharan & Kuruveettissery, 2025; Omar et al., 2017; Pohl et al., 2020). In addition, autistic children often experience co-occurring conditions, including behavioural disorders (e.g. oppositional defiant disorder) and mental health conditions (e.g. anxiety, depression) adding to the challenges of motherhood (Bearss & Kaat, 2020; Cochrane et al., 2022; Lai et al., 2019). As a result, mothers of autistic children report significantly higher stress and poorer mental health when compared to mothers of non-autistic children (AlTourah et al., 2020; Giallo et al., 2013; Santiago et al., 2024).

This heightened stress, combined with the increased demands faced by parents of autistic children, can contribute to relationship difficulties, such as interparental conflict (IPC; Westrupp et al., 2015). IPC refers to disagreements, tensions or hostile interactions between parents, which may involve verbal altercations (e.g. heated arguments, shouting, insults or threats) and/or physical confrontations (e.g. pushing, shoving or hitting) (Westrupp et al., 2015). Frequent and intense IPC can have a significant impact on family well-being and the mental health of all family members (Giallo, Seymour, Fogarty, et al., 2022). Within the general community, research consistently shows that IPC is associated with adverse outcomes for mothers, including poorer mental health and impacted parenting behaviours (e.g. lower warmth and acceptance) (Giallo, Seymour, Fogarty, et al., 2022; Kopystynska et al., 2022; Krishnakumar & Buehler, 2000; Lee & Lee, 2024). Elevated levels of IPC are also associated with increased emotional difficulties (e.g. depression, anxiety) and behavioural problems (e.g. conflict, aggression) in children across various developmental stages (Giallo et al., 2021; Harold et al., 2016; Jiang et al., 2025). The mother–child relationship itself can also be impacted, with emotional disruptions and impacts on attachment often resulting from ongoing conflict (Finger et al., 2009; Harold et al., 2016). These findings underscore the importance of understanding and addressing IPC to protect maternal and child mental health and promote healthier family dynamics.

Understanding the dynamics of IPC is particularly important in the context of mothers raising autistic children, as unique stressors faced by these mothers can significantly impact relationships. Research shows that mothers of autistic children often report significant strain on their couple relationship, leading to changes in how they interact and communicate with their partner (Goetz et al., 2019; Hock et al., 2012). As a result, mothers of autistic children report lower couple satisfaction (Brobst et al., 2009) and higher rates of separation (Hartley et al., 2010) when compared to mothers of non-autistic children. Despite this, research on IPC among mothers of autistic children remains limited. Of the available research, two studies recruited families from allied health services in the United States. In a cross-sectional study involving 183 mothers of autistic children, it was found that these mothers reported experiencing more severe and unresolved couple conflict than mothers of non-autistic children (Hartley et al., 2017). In another study of 174 mothers of autistic children it was found that these mothers reported experiencing more conflicts with their partner in daily life, particularly when their autistic child was present, compared to mothers of non-autistic children (Papp & Hartley, 2019). While important, this research remains limited, relying on families recruited from US allied health services, where the findings may not be generalisable to families from lower socioeconomic communities with restricted access to such services or to international contexts. In addition, the existing research uses cross-sectional designs, limiting our understanding of the long-term trajectories and lasting impacts of IPC on mothers.

Longitudinal studies in the general population show that IPC can continue across the parenting period for some mothers. In a longitudinal study of 4875 Australian families over 10 years (child ages 6–12 months to 10–11 years), four trajectories of IPC were identified: the majority (86.9%) of mothers reported consistently low IPC throughout the study; 8% experienced persistently elevated IPC; 3% had an increasing pattern of IPC over time; and 2% experienced a decrease in IPC over time (Giallo, Seymour, Fogarty, et al., 2022). Longitudinal research such as this has not been conducted with mothers of autistic children. This is needed given the unique stressors associated with raising an autistic child, such as increased caregiving demands, navigating support systems and societal stigma (Samuel et al., 2024; Schiltz et al., 2021), and the impact of these on parenting relationships over a long period of time. Understanding these trajectories is crucial to ensure early detection, monitoring and tailored support for families of autistic children, ultimately promoting family functioning and parental well-being.

Understanding the extent and course of IPC among mothers of autistic children is important, as ongoing conflict may impact their ability to cope and adapt to the stressors of parenthood. Mothers of autistic children often face heightened stress, poorer mental health and lower relationship satisfaction when compared with mothers of non-autistic children (AlTourah et al., 2020; Giallo et al., 2013; Sim et al., 2016). These factors can both contribute to and be exacerbated by IPC, with each potentially acting as mediators or covariates in shaping maternal well-being. Different conflict patterns (e.g. consistently high, intermittent or declining) may therefore influence maternal well-being in distinct ways. For example, persistently high IPC may worsen stress and reduce maternal well-being over time; while declining or intermittent conflict may allow mothers to maintain or improve well-being. Examining IPC trajectories and their link with maternal well-being is crucial for understanding differences in maternal outcomes and for designing effective support strategies for mothers raising autistic children.

The present study aimed to explore patterns of IPC over a 10-year period of child development for mothers of autistic children. The study had three aims:

1. To compare IPC over 10 years of child development between mothers of autistic and non-autistic children.
2. To identify subgroups of mothers of autistic children defined by their trajectories of IPC over 10 years of child development.
3. To examine the relationship between the distinct trajectories of IPC and mental health in mothers of autistic children, at child age 14–15 years.

## Methodology

### Study design and sample

Secondary data from the Growing Up in Australia: Longitudinal Study of Australian Children (LSAC) was used in this study. LSAC is a nationwide, ongoing study that tracks the development, well-being and social context of Australian children and their families (Department of Social Services et al., 2022). Initial ethics approval was granted by the Australian Institute of Family Studies Ethics Committee in 2004. Full information regarding the study design, sampling framework, procedures and data collection methods has been reported in a separate publication (Soloff et al., 2005). In brief, the LSAC dataset includes two cohorts: the Baby Cohort (B-cohort), with participants recruited between the ages of 6 and 12 months, and the Kindergarten Cohort (K-cohort), with participants recruited between the ages of 4 and 5 years. For both cohorts, a two-stage cluster sampling method was used. First, approximately 10% of Australian postcodes were selected, ensuring representation across states and urban versus rural areas. Then, children from each selected postcode were randomly chosen in proportion to the local population size, using data from Australia’s Medicare system.

Initiated in 2004, the study has completed 10 biennial follow-ups. All participants have been invited to participate in subsequent waves, where retention from Wave 1 to Wave 10 is 47.9%. At Wave 1, the B-cohort included 5107 infants (aged 3–12 months) and the K-cohort included 4983 children (aged 4–5 years), with cohort ages beginning to overlap from Wave 3. Data for this study are drawn from the B- and K-cohorts when child ages overlap at 4–5 years (B-cohort Wave 3, K-cohort Wave 1), 6–7 years (B-cohort Wave 4, K-cohort Wave 2), 8–9 years (B-cohort Wave 5, K-cohort Wave 3), 10–11 years (B-cohort Wave 6, K-cohort Wave 4), 12–13 years (B-cohort Wave 7, K-cohort Wave 5) and 14–15 years (B-cohort Wave 8, K-cohort Wave 6). Preliminary analyses found no significant differences between cohorts (Mohal et al., 2022), subsequently data from the B- and K-cohorts were merged. Retention rates were lower among families identifying as Aboriginal and Torres Strait Islander, those from non-English-speaking backgrounds, parents with lower levels of education and families living in rental housing (Sipthorp & Misson, 2007).

Child diagnosis of autism was provided by a single self-reported item (i.e. ‘Does study child have any of these conditions: Autism, Asperger’s, or other autism spectrum?’) by the child’s primary caregiver (~96% mothers), at any timepoint (child ages 6–7, 8–9, 10–11, 12–13 and 14–15 years). Information on autism diagnosis was not collected at the child age 4–5 years. A response of ‘Yes’ at any time point was interpreted as a lifetime presentation of autism, given its moderate stability as a diagnosis (Woolfenden et al., 2012).

A total of 346 autistic children were identified, with IPC data available for 333 (female and biological or adoptive) of their mothers across the six timepoints of interest. Of the non-autistic children, 8309 were identified, and IPC data was available for 8154 of their mothers. Demographic characteristics of the final sample are provided in Table 1; all data reported were collected at the first timepoint of interest (wave 3, B-cohort; wave 1, K-cohort). These are presented as reference indicators to characterise the sample, and there is likely to be change over the 10-year period for some of these characteristics. Independent sample *t* tests and chi-square (χ^2^) analyses conducted on continuous and categorical variables revealed that mothers of autistic children were significantly more likely to report that they were born in Australia (p < 0.001), were experiencing financial hardship (p < 0.01), had less children in the household (p < 0.001) and were less likely to be in full-time employment (p < 0.01) compared to mothers of non-autistic children.

**Table 1.** Child and mother demographic characteristics at child age 4–5 years.

Variable	Autistic children (n = 346) n (%)	Non-autistic children (n = 8309) n (%)
Child gender, male	254 (75.4)***	4106 (50.1)***
Child age (years), M (SD)	4.18 (0.39)	4.21 (0.41)
Age of diagnosis (months), M (SD)	62.85 (32.52)	-
Primary caregiver gender, female	121 (96.0)	4271 (97.6)
	Mothers of autistic children (*n* = 333) *n (%)*	Mothers of non-autistic children (*n* = 8145) *n (%)*
Age (years), M (SD)	34.37 (5.32)*	35.07 (5.12)*
Australia born	286 (85.9)***	6274 (77)***
Aboriginal or Torres Strait Islander	2 (0.6)	154 (1.9)
Regional, rural or remote area	119 (35.7)	2781 (34.2)
Experienced financial hardship	14 (4.2)**	148 (1.8)**
Socioeconomic status (SEIFA)	1009.55 (60.67)	1014.17 (60.56)
Education:		
Year 12 or above	67 (20.1)	2643 (32.4)
Year 11 or below	56 (16.8)	1698 (20.9)
Missing	210 (63.1)	3798 (46.6)
Full-time employed	182 (54.7)**	5109 (62.8)**
Mother relationship to study child:		
Biological	333 (100)	8125 (99.8)
Adopted	-	13 (0.2)
Step	-	7 (0.1)
Dual parent home	305 (91.6)	7631 (93.7)
No. of children in household, M (SD)	2.33 (0.87)***	2.5 (0.99)***

Demographics are presented for the first timepoint of interest (4–5 years), while some variables are static, some may change over time and are presented as a reference only.

Significant difference at *p < 0.05; **p < 0.01; ***p < 0.001.

### Measures

IPC was assessed at all timepoints using an adapted version of the IPC subscale from Ahrons’ (1981) Co-Parental Communication Scale (Department of Social Services et al., 2022). Mothers reported the frequency of verbal and physical conflict in their interactions with their partner through five items, rated on a 5-point frequency scale (i.e. 1 = never to 5 = always). Four items measured verbal conflict, focusing on disagreements, arguments, stress and anger (e.g. ‘We raise our voices when discussing issues related to our child’). A separate item assessed physical conflict, specifically addressing arguments involving physical confrontation or aggression (e.g. ‘We have physical fights in front of our child’). *Total IPC scores* were derived by summing all five items (range, 5–25), with higher scores indicating greater frequencies of IPC. This was calculated at each time point. Total IPC scores were used when analysing IPC trajectories and the relationship between IPC and mothers’ psychological distress. To descriptively report on the proportion of mothers experiencing high verbal or physical conflict at each timepoint, *high verbal IPC* was defined as those who responded ‘often’ or ‘always’ to at least one verbal item (Westrupp et al., 2015). While *high physical IPC* was defined as those who responded ‘sometimes’, ‘often’ or ‘always’ to the single physical item (Westrupp et al., 2015). The total IPC score has demonstrated strong internal consistency and robust construct validity (Giallo, Seymour, Fogarty, et al., 2022). Cronbach’s α for the current sample ranged from 0.68 to 0.77 across the timepoints of interest.

*Mothers’ psychological distress* was assessed when the children were 14 to 15 years, using the Kessler-6 (K-6; Kessler et al., 2002). Mothers reported the frequency of psychological distress using a six-item scale, rated on a 5-point scale (0 = none of the time to 4 = all of the time). Six items measured broad symptoms of nervousness, hopelessness, sadness and worthlessness. Scores were summed to generate a total score (range, 0–24), with higher scores indicating greater psychological distress. Cronbach’s α was 0.87 for mothers’ psychological distress when children were 14–15 years of age.

Measures used to assess demographic, interpersonal and broad social environmental characteristics are summarised in Table 2.

**Table 2.** Measures of interest.

*Construct*	Measure (source)	Additional information
*Demographic characteristics*		
Child characteristics		Gender, age, age of autism diagnosis, primary care giver gender.
Parent characteristics		Age, country of birth, Aboriginal or Torres Strait Islander Status, educational attainment, full-time employment, mothers’ relationship to study child, dual parent home, number of children in household.
*Interpersonal factors*		
Interparental conflict	Adapted Version of Co-Parental Communication Scale^a^	As above for interparental conflict.
Psychological distress	K-6^b^	As above for mothers’ psychological distress.
*Social environment factors*		
Remoteness of residence	Accessibility/Remoteness Index of Australia (ARIA)^c^	Measures distance from essential services on a 0–15 scale (0 = very accessible, 15 = very remote), grouped into five categories: Major cities, Inner regional, Outer regional, Remote and Very remote. For this study Inner regional, Outer regional, Remote Very remote were combined into ‘Regional, rural or remote area’.
Financial hardship	Hardship Scale^d^	Six items reported by the primary caregiver (e.g. ability to pay bills, rent/mortgage, heating/cooling, meals) rated dichotomously; higher scores indicate greater family financial hardship.
Relative socioeconomic position of residential area	Socio-Economic Index for Areas (SEIFA)^e^	Based on 2011 census data (including income, education, employment), scored with a mean of 1000 (SD = 100); lower scores indicate greater area disadvantage.

a Ahrons (1981).

b Kessler et al. (2002).

c Do and Care (2001).

d Bray (2001).

e Australian Bureau of Statistics (2011).

### Community involvement

Community members were not involved in any aspect of the research process.

### Data analysis and screening

Sample demographics and IPC for each time point were summarised using descriptive statistics in SPSS Version 24.0 (IBM Corp, 2016). Continuous data (i.e. total IPC scores) were analysed using independent sample *t* tests and categorical data (i.e. verbal and physical IPC categories) were analysed using chi-square (χ^2^) tests to explore differences between mothers of autistic children compared to mothers of non-autistic children (Aim 1).

Next, longitudinal latent class analysis (LLCA) was conducted using Mplus Version 8.7 (Muthén, 1998–2017) to identify subgroups of distinct IPC trajectories over six timepoints for mothers of autistic children (Aim 2). LLCA involved beginning with a one-class model and subsequently fitting models with increasing numbers of classes to identify the smallest number of classes that best fit the associations in the data. Given this is a data-driven approach, a range of criteria were considered when determining the best-fitting model (Weller et al., 2020). A better fitting model was indicated by a lower Likelihood Ratio Statistic (L2), Akaike Information Criterion (AIC) and Bayesian Information Criterion (BIC) value when comparing between models. In addition, an entropy value of > 0.80 was indicative of a model accurately classifying mothers into their most likely class. The improvement between neighbouring class models was determined by the Vuong-Lo-Mendell-Rubin Likelihood Ratio Test with a p-value < 0.05 indicative of a statistically significant improvement between class models. Finally, when deciding upon the final model, clinical utility and the number of participants in each class were considered.

Descriptive statistics examining the relationship between subgroups of IPC trajectories and mental health in mothers of autistic children were calculated using SPSS Version 24.0 (Aim 3). Class membership of IPC for mothers of autistic children was recorded and used to explore the association with mothers’ psychological distress. Continuous data (i.e. total K6 scores) were analysed using linear regression to compare psychological distress across IPC subgroups. Known confounders (i.e. child sex, maternal age, maternal country of birth, financial hardship, employment status and number of children in the house) were controlled for.

In relation to missing data, 9.3%–36.9% was missing for IPC across the six timepoints of interest. For Aims 1 and 3, multiple imputation was conducted to handle the missing data in SPSS. The results of the analysis were pooled across 50 parallel imputed datasets which incorporated variables that influence missingness (e.g. maternal country of birth, age, employment, indigenous status and socioeconomic position). The overall pattern of results between the multiple imputation and complete case data sets were similar, therefore only results for cases with imputed data are presented. For Aim 2, missing data were handled in Mplus using Full Information Maximum Likelihood (FIML). FIML uses all available data for individual cases to estimate model parameters and requires cases to have at least one variable of interest in the model to be estimated (Little et al., 2014).

## Results

### Aim 1: to compare IPC between mothers of autistic and non-autistic children

Table 3 presents the descriptive statistics for total IPC scores at each time point for mothers of autistic and non-autistic children. Descriptively, reports of IPC mean scores were highest at child ages 4–5 years for both groups of mothers. Mothers of autistic children were significantly more likely to report higher IPC mean scores when their children were aged 4–5, 6–7, 8–9 and 10–11 years compared to mothers of non-autistic children. For mothers of autistic children IPC was lowest at child age 14–15 years. For mothers of autistic children, the proportion of mothers reporting high verbal conflict peaked at child age 4–5 years. The proportion of mothers of autistic children reporting high verbal IPC at this timepoint was significantly higher than mothers of non-autistic children.

**Table 3.** Descriptive statistics for IPC scores at each wave for mothers of autistic and non-autistic children.

Timepoint/child age	Mothers of autistic children (n = 333)					Mothers of non-autistic children (n = 8154)				
	Descriptive statistics			High verbal IPC	High physical IPC	Descriptive statistics			High verbal IPC	High physical IPC
	Range	M	SD	n (%)	n (%)	Range	M	SD	n (%)	n (%)
1. 4–5 years	5–25	10.27***	2.85	39 (15.7)*	5 (1.9)	2–25	9.66***	2.58	707 (11.2)*	63 (0.9)
2. 6–7 years	4–22	9.77**	2.64	42 (14.5)	4 (1.3)	1–25	9.30**	2.62	771 (11)	59 (0.8)
3. 8–9 years	5–25	9.83*	2.80	36 (11.5)	1 (0.4)	3–25	9.38*	2.59	734 (10.3)	50 (0.8)
4. 10–11 years	5–20	9.86*	2.77	39 (13.9)	0 (0.0)	4–25	9.41*	2.58	758 (11.9)	32 (0.5)
5. 12–13 years	5–19	9.64	2.69	33 (11.6)	1 (0.4)	3–25	9.38	2.61	643 (10.1)	31 (0.6)
6. 14–15 years	5–21	9.63	2.59	28 (10.6)	2 (1.0)	4–23	9.40	2.61	577 (10.0)	38 (0.8)

Sample size varied across waves due to missing data.

Significant difference at *p < 0.05; **p < 0.01; ***p < 0.001.

### Aim 2: to identify groups of mothers of autistic children defined by IPC trajectories

LLCA was conducted to identify subgroups of mothers of autistic children defined by their trajectories of IPC over 10 years of child development. Model fit indices were inspected at classes 1 through to 5 (Table 4). The final model chosen was the two-class model. For this model, the AIC and BIC were lower than the one-class model, suggesting an improved fit. Entropy was approaching 0.80 and the average posterior probabilities (Class 1 = 94.9%, Class 2 = 89.6%) were high, indicating accurate classification of mothers. The Vuong-Lo-Mendell-Rubin statistic also indicated a significant difference between the one- and two-class models, suggesting that the two-class model gives a significant improvement in fit over the one-class model.

**Table 4.** Model fit indexes for latent classes of IPC for mothers of autistic children.

Model	L^2^	BIC	AIC	Entropy	Vuong-Lo-Mendell-Rubin	p-value
1-class	−3692.181	7454.059	7408.361	-	-	-
**2-class**	−**3482.144**	**7074.642**	**7002.287**	**0.764**	**1 vs 2 classes**	0.0021**
3-class	−3410.963	6972.938	6873.926	0.822	2 vs 3 classes	0.1704
4-class	−3372.138	6935.944	6810.276	0.868	3 vs 4 classes	0.3129
5-class	−3334.987	6902.299	6749.973	0.784	4 vs 5 classes	0.4152

Significant difference at *p < 0.05; **p < 0.01; ***p < 0.001.

**Figure 1.**
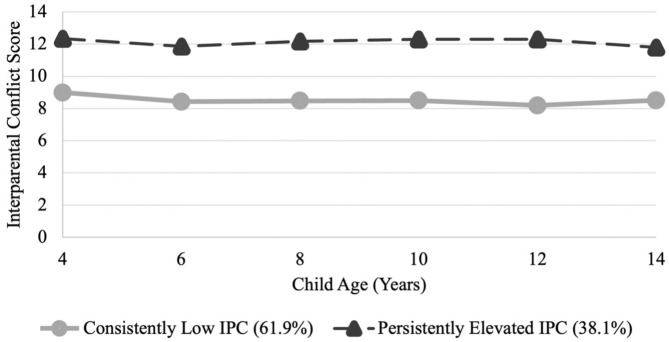
Trajectories of IPC across 10 years of child development for mothers of autistic children. Two distinct IPC patterns emerged: one group experienced consistently low IPC, while the other reported persistently elevated IPC.

Figure 1 presents the two-class model of IPC trajectories for mothers of autistic children. The majority of mothers were categorised into a class representing a trajectory of *consistently low* IPC across 10 years of child development (n = 206, 61.9%). While the second class represents mothers who experienced *persistently elevated* IPC across 10 years of child development (n = 127, 38.1%). Table 5 presents the IPC descriptives over time for mothers of autistic children in each class.

**Table 5.** Descriptive statistics for IPC across each class at each timepoint for mothers of autistic children.

Timepoint/child age	Class 1: Low IPC N = 206 M (SD)	Class 2: Elevated IPC N = 127 M (SD)
1. 4–5 years	8.99 (1.95)	12.34 (2.85)
2. 6–7 years	8.42 (1.78)	11.86 (2.84)
3. 8–9 years	8.47 (1.83)	12.17 (2.80)
4. 10–11 years	8.49 (1.76)	12.30 (2.77)
5. 12–13 years	8.19 (1.15)	12.29 (2.69)
6. 14–15 years	8.51 (1.91)	11.79 (2.59)

### Aim 3: IPC trajectories and psychological distress in mothers of autistic children

On average, both classes of mothers of autistic children reported moderate levels of psychological distress at child age 14–15 (Class 1: low IPC M = 4.23; Class 2: elevated IPC M = 5.69; standard deviations not produced for multiple imputed data). Linear regression analyses indicated that, after adjusting for confounding factors, mothers in the elevated IPC trajectory reported significantly higher psychological distress compared to mothers in the low IPC trajectory (B = 1.47, p = 0.011). Maternal age (B = −0.15, p = 0.010) and financial hardship (B = 0.72, p = 0.020) were also predictors of maternal psychological distress. Child sex, maternal employment, country of birth and number of children in the household were not significant predictors. These results show that IPC trajectory is a robust predictor of maternal psychological distress even when accounting for contextual factors.

## Discussion

The present study explored patterns of IPC over 10 years of child development for Australian mothers of autistic children, an area that remains largely under-researched.

### Aim 1: to compare IPC between mothers of autistic and non-autistic children

Mothers of autistic children were significantly more likely to report greater levels of IPC when their children were aged 4, 6, 8 and 10 years, compared to mothers of non-autistic children. This includes more frequent verbal altercations (e.g. arguments, shouting, insults or threats) and/or physical confrontations (e.g. pushing, shoving or hitting). These elevated levels of IPC may reflect the cumulative burden of caregiving demands, emotional stress and social isolation that can be associated with parenting an autistic child (Batra & Pandeya, 2023; Christopher et al., 2023; Devenish et al., 2020; Ebadi et al., 2021).

For mothers of autistic children, mean IPC scores and reports of high verbal conflict peaked when children were aged 4–5 years. This period coincides with the average age of autism diagnosis in Australia (Gibbs et al., 2019). Navigating the diagnostic process and uncertainty about a child’s future can increase tension, emotional burden and disrupt family dynamics (Feinberg et al., 2014; Filipova et al., 2024; Saini et al., 2015). This time is often marked by heightened maternal stress and strain on the couple relationship (Feinberg et al., 2014; Saini et al., 2015).

This period also coincides with many children starting kindergarten or transitioning to primary school, a time that introduces new challenges around structure, expectations and socialising (González-Moreira et al., 2024; Margetts, 2014). For mothers of autistic children, this period can be particularly challenging due to the need for increased advocacy, navigating complex support systems and managing their child’s behavioural and/or emotional difficulties. (Chen et al., 2020; Kim, 2023). These pressures can place significant strain on the couple relationship and intensify IPC (Hartley et al., 2018).

For mothers of autistic children, IPC mean scores were lowest when their children were aged 14–15 years. This may be due to adaption and coping over time, as by this stage, parents have often adjusted to their child’s needs, developed coping strategies and established routines that reduce conflict within the parenting relationship (Higgins et al., 2023; Iida et al., 2018).

### Aim 2: to identify groups of mothers of autistic children defined by IPC trajectories

Two distinct IPC trajectories emerged among mothers of autistic children. The majority (61.9%) followed a *consistently low* IPC trajectory across the 10-year period. This group may reflect families in which the couple relationship remains resilient despite the challenges of raising an autistic child. Although identifying protective factors was beyond this study’s scope, past research has identified that effective communication strategies (Banihashem et al., 2024), fair division of childcare (Newkirk et al., 2017) and perceived social support networks (Keneski et al., 2018) are factors that can reduce couple conflict.

A second class of mothers represented those who experienced *persistently elevated* IPC (38.1%) across 10 years of child development. These mothers may experience ongoing stress within the couple relationship, contributing to increased conflict. While not explored in this study, prior research has identified several factors that may explain higher conflict among mothers of autistic children. First, managing a child’s autism-related behaviours and attending to therapy or special education needs can be tiresome for parents (Haider, 2015; Samsell et al., 2021). Moreover, the constant pressure to meet these needs may lead to irritability and conflict between parents, as they may feel stressed and worn out (Hartley et al., 2017). In addition, the caregiving demands for an autistic child can fall unevenly on one partner, often the mother, leading to frustration and feelings of being overwhelmed (Hartley et al., 2014). This imbalance may cause resentment and conflicts between partners, particularly if one feels unsupported (Hartley et al., 2014). Furthermore, the cost of therapies, treatments and specialised education for an autistic child can create significant financial strain (Horlin et al., 2014; Uysal et al., 2025) which may lead to disagreements in the couple relationship. There are likely to be complex and often cumulative challenges faced by parents of autistic children which impact IPC and further research is warranted.

### Aim 3: IPC trajectories and psychological distress in mothers of autistic children

The relationship between IPC trajectories and maternal mental health in mothers of autistic children was examined when children were 14–15 years. Mothers experiencing *persistently elevated* IPC reported significantly greater psychological distress than those experiencing *consistently low* IPC. This finding supports prior research on community samples of mothers showing that ongoing conflict can lead to chronic stress, negatively impacting mothers’ mental health (Choi & Marks, 2008; Shrout et al., 2019). Persistently elevated IPC may also strain mothers’ coping skills, especially when raising an autistic child, further reducing coping capacity and impacting maternal well-being (Cuzzocrea et al., 2016). In addition, mothers in the persistently elevated group may have fewer chances to recover between conflicts, leading to emotional exhaustion over time and worsening of maternal mental health (Hartley et al., 2018). These findings highlight the negative impact of consistently elevated IPC on the mental health of the mothers of autistic children and stress the importance of tailored interventions that build coping strategies and reduce conflict in this group of mothers.

## Limitations and future directions

It is important to recognise the limitations of the current study as they provide important considerations for future research. The longitudinal cohort design relied on brief screening tools, limiting the depth of information that could be obtained. For example, the Co-Parental Communication Scale focused primarily on verbal and physical conflict, overlooking other forms such as emotional or psychological conflict (Zhu et al., 2010). The scale also included only one item on physical conflict, which may not capture the complexity or severity of physical altercations (Wangmann, 2011). Related to brief measures used in this study, autism status was identified using a single parent-report item, which may introduce bias due to caregiver knowledge, perception or willingness to disclose a diagnosis (Wang et al., 2022). Incorporating an evidence-based diagnostic tool would strengthen future analyses.

While maternal psychological distress was assessed using the Kessler-6 at each wave, the focus in this study was at a single timepoint, capturing distress over the previous month. As such, the K6 was used as a contemporaneous outcome rather than a longitudinal indicator of psychological distress. This measure is also likely to be shaped by broader contextual factors (e.g. financial hardship, family size, parenting challenges) in addition to IPC. Future research could therefore make use of repeated K6 data to examine the dynamic link between IPC and psychological distress as this would provide a clearer picture of their longitudinal relationship. Likewise, this study did not explore maternal, family or child factors that may shape IPC, such as challenging child behaviours or shifts in family relationships. Future research incorporating broader demographic, risk and protective factors would be valuable for identifying mothers most at risk of elevated IPC and informing the design and targeting of support services. In addition, changes in parental relationships over time were not examined. Given that interparental dynamics are likely to shift across time, future research could investigate how longitudinal changes in parental relationships intersect with trajectories of conflict and maternal well-being.

Generalisability is limited due to the underrepresentation of Aboriginal and Torres Strait Islander families, who experience unique stressors such as cultural dislocation, historical trauma and socioeconomic disadvantage (Ketheesan et al., 2020). Mothers born outside of Australia were also underrepresented. Greater inclusion of Indigenous and culturally diverse families is needed to better reflect varied experiences. This study also focused specifically on mothers of autistic children and did not compare them with other subgroups of caregivers (e.g. fathers, grandparents) or families (e.g. those of children with physical disabilities, chronic illness or from migrant backgrounds). Future research would benefit from examining a broader range of populations to provide a more comprehensive understanding of how child characteristics and family contexts shape IPC and maternal distress.

Use of secondary, de-identified LSAC data also prevented the inclusion of qualitative perspectives. Future research using mixed-methods or qualitative data collection would provide important insights into the lived experiences underlying these IPC trajectories and mother’s experiences of distress.

## Implications for interventions

This study highlights opportunities to support mothers of autistic children, particularly in the context of differing experiences of IPC over time. IPC peaked when children were aged 4–5 years, coinciding with the typical age of autism diagnosis in Australia, representing a crucial window for early intervention. Emotional support (e.g. counselling, peer networks) can help parents manage the stress of diagnosis and navigate early services (Drogomyretska et al., 2020; Ferrara & Esposito, 2017). Couple and parent-focused interventions that build communication, problem solving and coping skills may also help reduce conflict related to parenting a child with autism (Giallo, Seymour, Skinner, et al. 2022, 2024; Hock et al., 2022; Tellegen & Sanders, 2014). Moreover, clinicians working with newly diagnosed children are well positioned to identify relationship strain, provide timely support and offer appropriate referrals (Franzen & Mao, 2023; Hall & Graff, 2011).

The spike in IPC around ages 4–5 also aligns with the period when many children are attending kindergarten or preparing to transition to primary school, a time of heightened parental stress. This suggests that the early school years may represent a particularly vulnerable period for families in which additional stressors may heighten conflict. As such, advocacy training may be beneficial as it can empower parents with skills to communicate effectively with educators about their child’s needs, reducing parental stress (Burke, 2013). Strengthening family-school collaboration can further enhance communication, align expectations and reduce parental stress (Hsiao et al., 2017; Schultz et al., 2016).

The identification of two distinct IPC trajectories among mothers of autistic children also underscores that families are not homogeneous in their experiences of conflict. The existence of a sizable subgroup of mothers experiencing sustained conflict over a decade suggests the need for support that can both prevent the escalation of conflict and provide long-term assistance for families. This is especially important as mothers in the *persistently elevated* IPC group reported significantly greater psychological distress compared to those experiencing *consistently low* IPC. While causal pathways cannot be inferred, this finding underscores the need for targeted support and suggests that interventions aimed at reducing IPC may also have potential to support maternal mental health. Previous research has shown that interventions such as conflict resolution therapy and coparenting workshops can directly address chronic conflict and support maternal mental health (Askari et al., 2013; Cooke et al., 2023; Feinberg & Kan, 2008; Stefaniuk & Fahami, 2024). The cumulative impact of ongoing, unresolved conflict further underscores the importance of sustained family focused interventions and ongoing follow-up with high-risk families (Haight et al., 2023).

These implications are illustrative examples of potential interventions. However, the results highlight the importance of further investigation of individual, family and child-level factors that shape IPC trajectories and that place some mothers on trajectories of elevated IPC. Addressing these questions represents an important area for future research.

## Conclusion

In conclusion, this study provides valuable insights into the patterns of IPC experienced by Australian mothers of autistic children over a 10-year period. The findings highlight distinct trajectories of IPC, with most mothers experiencing *consistently low* IPC, while a smaller group face *persistently elevated* levels of IPC across their child’s development. Fluctuations in IPC were linked to key developmental stages in a child’s life, particularly during the critical period of autism diagnosis and the transition to school. The study also emphasised the impact of IPC on maternal mental health, showing that *persistently elevated* IPC can contribute to greater psychological distress compared with experiences of *consistently low* IPC. These findings are essential for creating tailored and effective strategies to support mothers raising autistic children. Early intervention for IPC can help reduce the risk of more serious family conflict, ultimately fostering safer family environments and improving maternal well-being. To achieve this, it is crucial that support services account for the unique needs of mothers raising autistic children.
